# Comparison of Tongue Acupuncture and Traditional Acupuncture in the Treatment of Poststroke Dysarthria: A Meta-Analysis and Tongue Acupuncture System Classification Evaluation

**DOI:** 10.1155/2022/4897863

**Published:** 2022-11-01

**Authors:** Xinming Yang, Lei Shi, Dawei Ran, Ying Kong, Weiping Shi, Jiaxiao Zhou, Huasong Gao, Yutong Han, Huixue Zhang

**Affiliations:** ^1^First Teaching Hospital of Tianjin University of Traditional Chinese Medicine, Tianjin, China; ^2^National Clinical Research Center for Chinese Medicine Acupuncture and Moxibustion, Tianjin, China

## Abstract

**Objective:**

To discuss whether tongue acupuncture is more effective than traditional acupuncture in the treatment of poststroke dysarthria and explore the advantage of tongue acupuncture treatment parameters.

**Methods:**

We evaluated the efficacy of tongue acupuncture compared with traditional acupuncture through a rigorous meta-analysis process. The included studies were from eight databases in English and Chinese. The Cochrane risk of bias assessment tool was used to evaluate the quality of studies. Stata15.1 software was used for meta-analysis and sensitivity analysis. Tongue acupuncture therapeutic parameters were classified and counted based on tongue acupoint location, acupuncture manipulation, and the number of manipulations. Subgroup analysis was used to compare the differences between various treatment parameters. *Outcome* The meta-analysis eventually included a total of 9 studies. Tongue acupuncture is superior to traditional acupuncture in clinical efficacy [OR = 3.62, 95%Cl (2.24, 5.85), *P* < 0.0001], FDA score [SMD = −1.99, 95%Cl (−3.77, −0.21), *P*=0.028], and NIHSS score [WMD = 0.86, 95%Cl (0.15, 1.57), *P*=0.017, I2 = 31.7%] in the treatment of poststroke dysarthria. According to the classified statistics of tongue acupuncture treatment parameters, there are three kinds of tongue acupuncture points in 9 studies: lingual surface, sublingual, and both lingual surface and sublingual acupoints. The operation methods include the oblique stabbing of the root of the tongue, twisting after stabbing, and acupoint pricking. The number of operation methods varies from 1 to 3.

**Conclusion:**

Tongue acupuncture outperforms traditional acupuncture in terms of clinical efficacy, FDA score, and NIHSS score in the treatment of poststroke dysarthria. The curative effect of sublingual acupoints is better than that of lingual surface acupoints, the combined use of multiple manipulations is better than that of a single manipulation, and acupuncture manipulation has a cumulative effect. PROSPERO registration number: CRD42021285722.

## 1. Introduction

Dysarthria, also known as dyskinesia dysarthria, is a speech disease characterized by muscular paralysis and dyskinesia of the articulatory organs caused by lesions of the central, peripheral nervous system, or the muscle itself. It is one of the common complications of a stroke [1]. The main symptoms of dysarthria patients include unclear speech, long tone, dysrhythmic control of speech speed, monotonous language, and silence. It is often accompanied by dysphagia, increased sputum and salivation, and other symptoms [2]. About 20%–30% of stroke patients will experience dysarthria, and most of them will have psychological obstacles [3]. It is vital to carry out rehabilitation treatments for dysarthria patients.

Speech training is the main treatment for postapoplectic dysarthria [4, 5]. Breathing and rhythm training, articulation training, and training to overcome nasal breath and laborious sounds are all part of speech training [6]. Other treatment methods include acupuncture remedy [7, 8], massage manipulation therapy, pronunciation EMG stimulation therapy, hyperbaric oxygen therapy (HPO), and training of articulatory organs.

In China's domestic research during the last ten years, clinical trials of acupuncture in the treatment of dysarthria after stroke have yielded favorable curative effects. The therapeutic acupoints REN23 and GB20 are distributed in the glossopharyngeal, sublingual, and vagal innervation areas. Acupuncture can improve nerve sensitivity and promote the recovery of motor function. In recent years, studies have frequently reported on the comparison of curative effects between different acupuncture points to explore better acupoint selection methods. Tongue acupuncture in the treatment of dysarthria is a new method of acupoint selection that improves the original traditional acupoint selection method by increasing the number of tongue points and manipulation. Acupuncture on the tongue can increase central nervous system excitability and activate the functional activity of the language area of the brain.

Previous studies have proved that electroacupuncture combined with language training and manipulation is better than simple language training [7]. The results of its subgroup analysis of manipulation types suggested that there was no significant difference between different manipulations. Through this meta-analysis, we intend to provide evidence that the curative effect of tongue acupuncture is better than traditional acupuncture and to judge whether different acupoint prescriptions have relevant effects on the curative effect, so as to provide future direction of acupuncture for clinical in poststroke dysarthria research.

## 2. Methods

This systematic review and meta-analysis were conducted based on the preferred reporting items for systematic review and meta-analysis protocols (PRISMA-P) [9], and The Cochrane Handbook for Systematic Reviews of Interventions [10]. This study has been registered on PROSPERO with registration number CRD42021285722.

### 2.1. Patient and Public Involvement

Since the article is a systematic evaluation based on the published original research, it does not involve patients and the public.

## 3. Searching Strategies

We searched eight databases in China and overseas, including four in English: PubMed, Embase, Cochrane Library, and Web of Science, and four in Chinese: CNKI, Wanfang database, Chongqing VIP database, and China Biology Medicine disc (CBMdisc). Two researchers conducted the retrieval process, and the final retrieval formula was determined by consultation. The search scope was from the beginning of database construction to December 2021. the keywords in PubMed include “Dysarthrias,” “Dysarthosis,” “Dysarthoses,” “Dysarthria, Spastic,” “Dysarthria, Mixed,” “Dysarthria, Scanning,” “Dysarthria, Flaccid,” “Dysarthria, Guttural,” “Stroke,” “Strokes,” “Cerebrovascular Accident,” “CVA,” “Cerebrovascular Apoplexy,” “Vascular Accident, Brain,” “Cerebrovascular Stroke,” “Apoplexy,” “Cerebral Stroke,” “acupuncture,” “electroacupuncture,” and “Pharmacopuncture.” Table 1 shows the retrieval in PubMed (Table 1).

### 3.1. Inclusion/Exclusion Criteria

#### 3.1.1. Study

The studies included in our systematic evaluation were clinical randomized controlled trials of tongue acupuncture in the treatment of poststroke dysarthria. Nonrandomized controlled trials, semirandomized controlled trials, animal experiments, medical record reports, systematic evaluation or review, qualitative studies, conference reports, and graduation theses were excluded.

#### 3.1.2. Participants

The subjects were patients with dysarthria after stroke, including patients with intracerebral hemorrhage and cerebral infarction. The criteria for exclusion included the following: (1) abnormal function caused by abnormal morphology of articulation organs; (2) complete aphasia; and (3) dysarthria not caused by cerebral apoplexy.

#### 3.1.3. Interventions and Controls

The intervention group selected tongue acupuncture. The intervention groups had more acupoints on the tongue and more complex operation methods than the control group.

The control group selected traditional acupuncture. It means that the acupoints selected for treatment come from the mainstream treatment methods for poststroke dysarthria in textbooks or guidelines. Moreover, due to the particularity of stroke patients, patients need basic treatment during the clinical trial. To avoid bias, we included the clinical trial of unified basic therapy in both the treatment and control groups.

#### 3.1.4. Outcomes

The curative effect was our primary outcome measure. The secondary outcome measure was the modified Frenchay dysarthria assessment scale score (FDA) and the NIHSS score.

### 3.2. Data Extraction

We made a detailed information extraction table using Microsoft Word, including the study's basic information, research object information, diagnostic criteria, the number of intervention groups and control groups, intervention methods, outcome indicators, follow-up time and measurement methods, analysis and statistics of results, and adverse events. Two researchers carried out the entire information extraction process back-to-back and the disputed items were discussed to determine the outcome after discussion. Finally, the main information of the study was summarized in a short table for display.

### 3.3. Risk of Bias Assessment

The Cochrane bias risk assessment tool [11] was used to conduct the quality evaluation, which covers selection bias, performance bias, attrition bias, reporting bias, and other biases. The tool categorizes bias into three categories: “low risk,” “high risk,” and “unclear.” Risk assessment was conducted by two researchers and any disputes were resolved through discussion.

### 3.4. Data Analysis

Stata15.1 software was used for data synthesis analysis. We choose the odds ratio (OR) with a 95% confidence interval (CI) as the scale index for dichotomous outcomes (Clinical efficacy). Standardized mean difference (SMD) with 95% CI was selected as the index for the modified Frenchay dysarthria assessment scale score. Mean difference (MD) with 95% CI was selected as the index for the NIHSS score. And a heterogeneity test was conducted among the studies, with the I^2^ statistics [12]. The fixed effect model was utilized if the heterogeneity result was *P* ≥ 0.1 and I2 ≤ 50%; otherwise, the random effect model was used. We also examined the impact of a single study on the overall meta-analysis through sensitivity analysis. The detection of publication bias was not performed since the number of included studies was fewer [10].

## 4. Results

**Figure 1:**
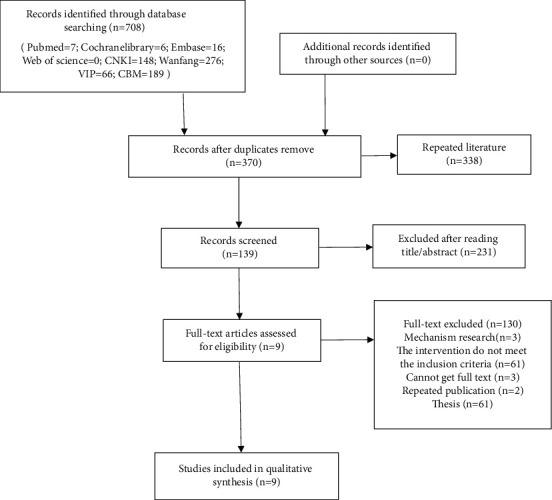
The whole screening process of articles.

A total of 708 articles were included in the preliminary screening stage after searching 8 databases. We eliminated 338 duplicate articles and 231 irrelevant articles by reading titles and abstracts. 139 articles entered the fine screening stage of full-text reading. Finally, according to the inclusion and exclusion criteria of this study, 9 clinical studies were included for qualitative analysis. The whole screening process is shown in Figure 1.

### 4.1. Characteristics of the Included Trials

The important information of the 9 studies finally included is shown in Table 2.

#### 4.1.1. Participants

The study comprised of 565 patients with poststroke dysarthria, with 286 receiving tongue acupuncture and the remaining 279 receiving traditional acupuncture. All nine studies were conducted in China. The age of onset was mainly about 60 years old, and the incidence rate of males was higher than that of females.

#### 4.1.2. Interventions and Controls

All participants of experimental groups selected tongue acupuncture. The acupoints were mostly located under the tongue or on the surface of the tongue in the selected 9 trials. The majority of the acupuncture methods involved oblique stabbing of the root of the tongue and then twisting the needle body, or pricking and bleeding on the selected acupoints. All the participants of the control group selected traditional body acupoints. The average needle retention time was 30 minutes and the treatment course ranged from a month to two months.

#### 4.1.3. Outcome Measures

Clinical efficacy was reported in all nine studies, with six using the Frenchay dysarthria rating scale and two using the NIHSS score.

### 4.2. Risk of Bias in Individual Trials

**Figure 2:**
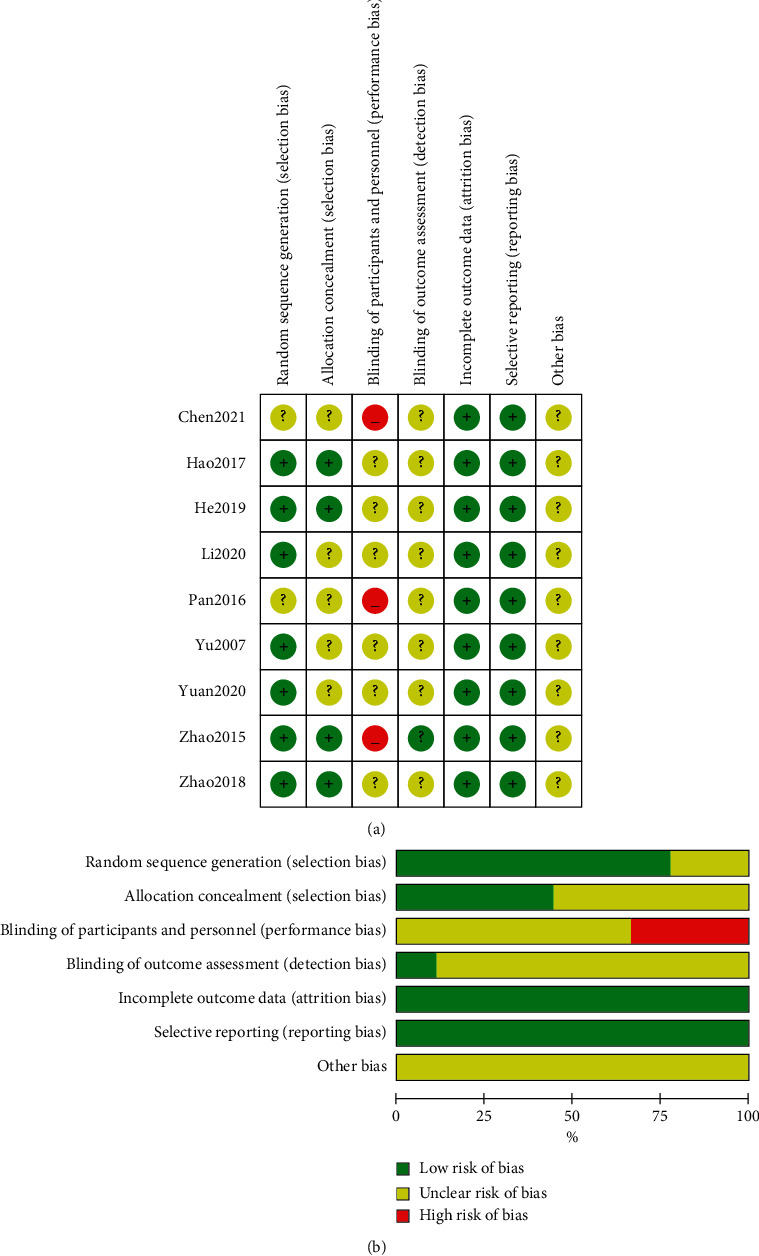
Risk of bias graph and summary.

Two of the nine included studies' randomization was unclear. Three trials were classified as 'high risk' because participants were not blinded. Only one research explicitly described the use of the blind in data assessment, which was not mentioned in other studies. There was no attrition bias and reporting bias in all studies (Figure 2).

### 4.3. Analysis of Data

#### 4.3.1. Clinical Efficacy

**Figure 3:**
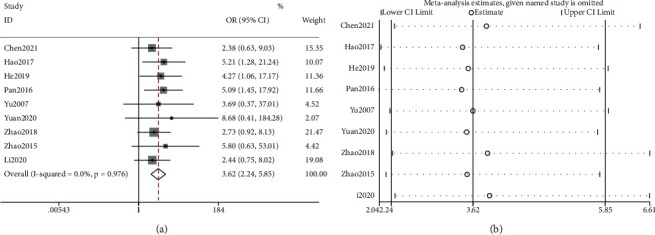
Data analysis of the curative effect: (a) meta-analysis of clinical efficacy and (b) sensitivity analysis.

Clinical efficacy was reported in all 9 studies. Meta-analysis showed that tongue acupuncture was superior to traditional acupuncture in the treatment of poststroke dysarthria [OR = 3.62, 95%Cl (2.24, 5.85), *P* < 0.0001, I2 = 0.0%] (Figure 3(a)). The homogeneity of the included studies was high and the fixed effect model was used. The sensitivity analysis shows that the analysis model is relatively reliable (Figure 3(b)).

#### 4.3.2. The Modified Frenchay Dysarthria Assessment Scale Score (FDA)

**Figure 4:**
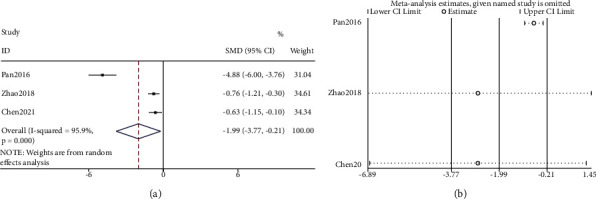
FDA score data analysis: (a) FDA score meta-analysis and (b) sensitivity analysis.

Only 3 studies provided FDA score data. Because of the differences in the FDA scores of the three trials, SMD (standardized mean difference) was used as the evaluation index to reduce the impact of different dimensions. Data were pooled using a random effects model. Meta-analysis of FDA scores in 3 studies showed that tongue acupuncture was better than traditional acupuncture in improving FDA scores [SMD = −1.99, 95% Cl (−3.77, −0.21), *P*=0.028, I2 = 95.9%] (Figure 4(a)). Sensitivity analysis revealed that no study had a significant impact on the analysis results (Figure 4(b)).

#### 4.3.3. The NIHSS Score

WMD was chosen as the evaluation index after combining the data from only two studies that reported NIHSS scores. In addition, since the heterogeneity evaluation was less than 50%, the fixed effect model was selected. The result showed that the tongue acupuncture group was better than traditional acupuncture group in improving NIHSS score [WMD = 0.86, 95% Cl (0.15, 1.57), *P*=0.017, I2 = 31.7%].

### 4.4. Classification Summary and Subgroup Analysis

#### 4.4.1. Classification Summary

**Figure 5:**
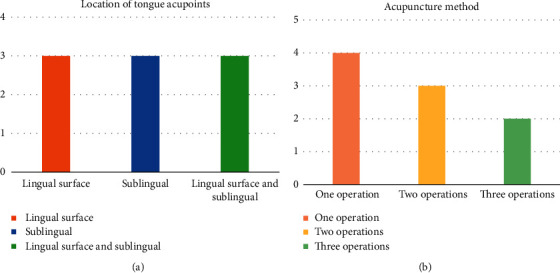
Study number of acupoint selection and operation.

We classified and summarized the included trials (Table 3). According to the different positions of acupoints, the included studies were divided into three categories: lingual surface, sublingual, and both lingual surface and sublingual. Additionally, according to the different operation methods of acupuncture, the research was divided into three categories: oblique stabbing into the tongue root, twisting the needle body after stabbing, and acupoints pricking. Among them, four studies used only one operation method, three studies used two, and two studies used three at the same time. The count statistics of the studies are shown in Figure 5.

#### 4.4.2. Subgroup Analysis

Subgroup analysis was performed based on the classification of 9 studies to determine if tongue acupuncture with varied acupoint locations and different number of operation methods was superior to the traditional acupuncture group. And we also compared the differences among the different subgroups. The results are shown in Table 4.

In the subtypes of different positions of acupoints, the curative effects of the lingual surface group, the sublingual group, and the lingual surface combined with the sublingual group were better than those of the traditional acupoint selection group. Through the comparison of *P* values among the three groups, the effect of sublingual group [OR = 3.74, 95% Cl (1.70, 8.22), *P*=0.001, I2 = 0.0%] and lingual surface combined with sublingual group [OR = 3.82, 95% Cl (1.77, 8.24), *P*=0.001, I2 = 0.0%] is better than that of simple lingual surface group [OR = 3.17, 95% Cl (1.19, 8.45), *P*=0.021, I2 = 0.0%].

Among the subgroups with different number of operation methods, the three subgroups were statistically significant, and the curative effect of the experimental group was better than that of the control group. By comparing the *P* values of the three groups, the subgroups using two operation methods [OR = 3.87, 95% Cl (1.67, 8.95), *P*=0.002, I2 = 0.0%] or three operation methods [OR = 4.71, 95% Cl (1.75, 12.66), *P*=0.002, I2 = 0.0%] were better than the subgroups using only one operation method [OR = 2.97, 95% Cl (1.43, 6.15), *P*=0.003, I2 = 0.0%].

### 4.5. Adverse Event

Acupuncture caused no serious adverse events in any of the nine studies included.

## 5. Discussion

### 5.1. Acupuncture for the Poststroke Dysarthria

The mechanism of acupuncture for poststroke dysarthria may be considered from the recovery of central and peripheral nerve functions. According to the current research, it involved the bidirectional regulation of acupuncture on inflammatory cells after stroke, regeneration, and promotion of nerve and blood vessels at the injured site, blood flow regulation at the ischemic site, and cerebral edema improvement. [22, 23] A study has shown that acupuncture can reduce poststroke brain edema and promote the proliferation, migration, and differentiation of neural stem cells [24], and can promote the generation of neurotrophic factors, so as to protect nerves [25]. Furthermore, it can regulate cerebral blood flow and activate cerebral angiogenesis after ischemic cerebral infarction [26]. For the peripheral nerve, it has been proved that acupuncture can promote the functional recovery of nerves and muscles for upper limb nerve injury [27, 28]. Furthermore, acupuncture may promote facial nerve regeneration by upregulating the expression of GDNF and N-cadherin mRNA in facial neurons, which demonstrates acupuncture's nerve regeneration-promoting function [29].

In the treatment of dysarthria, many sound therapies, including yawning sighs, resonance sound therapy, visual and EMG biofeedback, progressive relaxation, and perilaryngeal massage, can relieve or rebalance laryngeal muscle hyperfunction and alleviate symptoms [30–32]. Perilaryngeal treatment can improve dysphonia caused by increased muscle tone [33]. Acupuncture at relevant acupoints can shorten the distance between the hyoid bone and thyroid cartilage [34], which can alleviate the tension and spasm of neck muscles. This also provides evidence of curative effects for the treatment of dysarthria with tongue acupuncture. Most of the acupoints in tongue acupuncture therapy involve the spasm muscles of the neck area but traditional acupuncture did not realize this. This may be the reason why tongue acupuncture is superior to traditional acupuncture.

### 5.2. Interpretation of Results

Based on the results of this meta-analysis, tongue acupuncture was shown to be more effective than traditional acupuncture in the treatment of poststroke dysarthria. Tongue acupuncture is superior to traditional acupuncture in efficacy index, FDA score, and NIHSS score. It indicates that tongue acupuncture is more effective than traditional acupuncture at restoring language and nerve function in patients with poststroke dysarthria.

Through the analysis of the subgroups of the tongue acupuncture group in the acupoint position selection, we conclude that the curative effect of sublingual acupoint selection or the lingual surface combined with the sublingual acupoint selection group is better than that of the traditional point selection group. It is suggested that sublingual acupoint selection has a better effect than lingual surface acupoint selection and that lingual surface and sublingual combined acupoint selection has an effect similar to simple sublingual acupoint selection. The results imply that sublingual acupoint selection has the advantage of a curative effect in clinics.

The curative effects of the three groups are better than the control group in the subgroup analysis of the number of acupuncture operation methods but the analysis results suggest that using more than two methods is better than using only one method. The effect of using two method groups is similar to using three method groups. It can be considered that an appropriate number of acupuncture manipulations can improve the curative effect, and the manipulation has a cumulative effect.

### 5.3. The Control of Study Homogeneity and Evaluation Basis of Efficacy Index

To ensure the consistency and analyzability of the included studies, we strictly selected the control group and the experimental group of the included studies. All included studies were the comparison between tongue acupuncture and traditional acupuncture. In the tongue acupuncture group, the acupoints on the surface of the tongue or under the tongue were selected. The acupoints in the traditional acupuncture group were chosen from those recommended in textbooks or guidelines, such as body acupuncture, scalp acupuncture, and neck acupuncture. To ensure that the difference in curative effect only comes from tongue acupuncture, the studies we included ensure the consistency of basic treatment. Moreover, the difference in treatment between the experimental group and the control group is only in the acupoint selection and acupuncture manipulation.

The Frenchay dysarthria rating scale assigned a total score of 28 points based on reflex, respiration, lip, jaw, soft palate, throat, tongue, and speech. Recovery: the speech function reached grade 5, with a score of 28∼27. Remarkable effect: the speech function evaluation was improved by 2∼4 grades, with a score of 26∼18. Improvement: the evaluation of speech function was improved by 1 grade, with a score of 17∼14; Invalid: there was no change in speech function evaluation, and the score was 6∼0.

### 5.4. Research Evaluation and Prospects

The limitation of this study is that only 9 studies were included, and all of them had the limitations of small sample size and lack of rigorous research methods. The observation index was single, only curative effect Frenchay score, and NIHSS score. The safety issues of the included studies were not reported in detail and the safety assessment was not considered. The advantage of this study is that it will further classify and evaluate the tongue acupuncture group's acupoint selection and operation methods, as well as strictly control the intervention methods included in the study.

To improve the clinical evidence of tongue acupuncture in the treatment of poststroke dysarthria and to perfect the parameters of acupuncture, researchers can focus on the superior efficacy of tongue acupuncture, expand the sample size, improve the research methods, and explore the superior acupoint selection and acupuncture manipulation in future clinical research. Moreover, the research on the mechanism of acupuncture in the treatment of poststroke dysarthria is still in the research blind area. More focus should be given to fundamental research in this area.

## 6. Conclusion

This meta-analysis shows that tongue acupuncture is superior to traditional acupuncture in terms of clinical efficacy, FDA score, and NIHSS score in the treatment of poststroke dysarthria. The effect of acupoints selection of sublingual is better than that on the lingual surface. Also, the results suggested that the more acupuncture manipulation were used, the better were the results. In clinical practice, tongue acupuncture (especially sublingual acupoint selection) can be added to treat poststroke dysarthria, and the use of acupuncture manipulation can be increased. The actual efficacy and better parameters of tongue acupuncture need to be confirmed by large samples and more rigorous clinical trials, and the clinical safety also needs more experiments to evaluate.

## Figures and Tables

**Table 1:** Retrieval for PubMed.

^#^1	((((((((((((((((((((((Dysarthrias [Title/Abstract]) OR (Dysarthosis [Title/Abstract])) OR (Dysarthoses [Title/Abstract])) OR (dysarthria, Spastic [Title/Abstract])) OR (dysarthrias, Spastic [Title/Abstract])) OR (spastic Dysarthria [Title/Abstract])) OR (spastic Dysarthrias [Title/Abstract])) OR (dysarthria, mixed)) OR (dysarthrias, mixed)) OR (mixed dysarthria)) OR (mixed dysarthrias)) OR (dysarthria, scanning)) OR (dysarthrias, scanning)) OR (scanning dysarthria)) OR (scanning dysarthrias)) OR (dysarthria, flaccid)) OR (dysarthrias, flaccid)) OR (flaccid dysarthria)) OR (flaccid dysarthrias)) OR (dysarthria, guttural)) OR (dysarthrias, guttural)) OR (guttural dysarthria)) OR (guttural dysarthrias).
^#^2	((((((((((((((((((((Stroke [Title/Abstract]) OR (Strokes [Title/Abstract])) OR (cerebrovascular Accident [Title/Abstract])) OR (cerebrovascular Accidents [Title/Abstract])) OR (CVA [Title/Abstract])) OR (CVAs [Title/Abstract])) OR (cerebrovascular Apoplexy [Title/Abstract])) OR (apoplexy, Cerebrovascular[Title/Abstract])) OR (vascular accident, brain)) OR (brain vascular accident)) OR (brain vascular accidents)) OR (vascular accidents, brain)) OR (cerebrovascular stroke)) OR (cerebrovascular strokes)) OR (stroke, cerebrovascular)) OR (strokes, cerebrovascular)) OR (apoplexy)) OR (cerebral stroke)) OR (cerebral strokes)) OR (stroke, cerebral)) OR (strokes, cerebral).
^#^3	“acupuncture” [Title/Abstract] OR “electroacupuncture” [Title/Abstract] OR “Pharmacopuncture” [Title/Abstract].
^#^4	^#^1 and ^#^2 and ^#^3.

**Table 2:** A summary of key information of the included studies.

Study (Years)	Patients (*N*)	Characteristics of patients (Age = years, disease course = days)	Diagnostic criteria	Intervene of the tongue acupuncture group	Intervene of the traditional acupuncture group	Outcome measures	Adverse events
Chen et al. (2021) [13]	58 EG: 29 CG: 29	EG : Male: 17 female: 12 Age: 62.87 ± 8.66 Disease course: 24.24 ± 1.19 CG : Male: 11 female: 18 Age: 63.00 ± 6.50 Disease course: 23.54 ± 1.25	The 4th national symposium on cerebrovascular diseases of the Chinese medical association Frenchay dysarthria rating scale	Acupoint: tongue acupoint (shengen), body acupoints (LU7, KID6, HT5, PC6, ST40, SP6, LI4, LIV3, GB20) TA position: both sides of the sublingual frenum TA operation: Oblique thorn 0.8 inches to the root of the tongue and twist the needle 20 min. 5 times per week; 6 weeks	Acupoint: jinjin and yuye bloodletting, body acupoints (LU7, KID6, HT5, PC6, ST40, SP6, LI4, LIV3, GB20) 3 times per week; 6 weeks	Clinical efficacy; Frenchay dysarthria rating scale	Not occurred

Hao et al. (2017) [14]	60 EG: 30 CG: 30	EG : Male: 21 female: 9 Age: 62.3 (54.3∼70.3) Disease course: 5.6 months CG : Male: 24 female: 6 Age: 64.5 (56.8∼72.2) Disease course: 5.6 months	The 4th national symposium on cerebrovascular diseases of the Chinese medical association Frenchay dysarthria rating scale	Acupoint: tongue acupoint (bilateral beside REN23, above REN23, jinjin, yuye) TA position: Pang Lianquan, Shang Lian Quan: upper edge of thyroid cartilage; Jinjin, yuye: Both sides of the sublingual frenum TA operation: Oblique thorn(45°–60°) 25–40 mm to the root of the tongue and twist needle 20 s 30 min, 6 times per week; 4 weeks	Acupoint: REN23, DU15 6 times per week; 4 weeks	Clinical efficacy	Not reported

He et al. (2019) [15]	70 EG: 35 CG: 35	EG : Male: 15 female: 20 Age: 61.20 ± 10.27 Disease course: 10.57 ± 6.06 CG : Male: 17 female: 18 Age: 57.86 ± 12.11 Disease course: 9.54 ± 6.19	Guidelines for prevention and treatment of cerebrovascular diseases in China Frenchay dysarthria rating scale	Acupoint: tongue acupoint (shengen, bilateral zuoliang, bilateral zhimai, jinjin, yuye, tongue prick); body acupoints: GB20, HT5, SP6, REN23 TA position: shengen, zuoliang, zhimai: sublingual; TA operation: oblique thorn< 1 inch to the root of the tongue and twist needle 30 min, 1 time per day; 20 days	Body acupoints: GB20, HT5, SP6, REN23 1 time per day; 20 days	Clinical efficacy;	Not reported

Li et al. (2020) [16]	75EG: 38 CG: 37	EG : Male: 25 female: 13 Age: 62.00 ± 8.37 Disease course: 6.92 ± 3.12 months CG : Male: 27 female: 10 Age: 60.19 ± 8.84 Disease course: 6.95 ± 3.16 months	Guidelines for prevention and treatment of cerebrovascular diseases in China Frenchay dysarthria rating scale	Acupoint: tongue acupoint (juquan, tongue surface prick); body acupoints: DU26, REN23, GB20, SJ17, jinjin, yuye, posterior pharyngeal wall. TA position: juquan: center of lingual surface TA operation: acupoints prick 30 min, 5 times per week; 3 weeks	Body acupoints: DU26, REN23, GB20, SJ17, jinjin, yuye, posterior pharyngeal wall	Clinical efficacy; NIHSS	Not reported

Pan et al. (2016) [17]	50 EG: 25 CG: 25	EG : Male: 12 female: 13 Age: 50.5 ± 0.7 Disease course: 30 ± 0.5 CG : Male: 12 female: 13 Age: 50.8 ± 0.5 Disease course: 30 ± 0.8	Frenchay dysarthria rating scale	Acupoint: Tongue acupoint (haiquan, juquan); body acupoints: Du meridian acupoints on the head TA position: haiquan: root of lingual frenum; juquan: center of the lingual surface TA operation: feed the needle 1.2–1.5 inches and twist for 10 s 30 min, 1 time per day; 2 months	Body acupoints: Du meridian acupoints on the head, 30 min 1 time per day; 2 months	Clinical efficacy; Frenchay dysarthria rating scale	Not reported

Yu et al. (2007) [18]	82 EG: 44 CG: 38	EG : Male: 26 female: 18 Age: 40∼79 Disease course: 20∼180 CG : Male: 23 female: 15 Age: 42∼80 Disease course: 20∼180	The 4th national symposium on cerebrovascular diseases of the Chinese medical association diseases in China	Acupoint: tongue acupoint (xinxue, pixue); Body acupoints: Jinjin, yuye, GB20, REN23, HT5, PC6). TA position: xinxue: the tip of the tongue; pixue: center of the lingual surface TA operation: fast needle feeding and twist 10 times 20 min, 1 time per day; 1 month	Body acupoints: Jinjin, yuye, GB20, REN23, HT5, PC6) 1 time per day; 1 month	Clinical efficacy	Not reported

Yuan et al. (2020) [19]	30 EG: 15 CG: 15	Male: 17 female: 13 Age: 64.35. Disease course: 10∼90	Frenchay dysarthria rating scale	Acupoint: tongue acupoint (juhou); TA position: juhou: tingual surface TA operation: oblique stab into the root of the tongue 30 min, 1 time per day; 10 times a course of treatment	Body acupoints: Jinjin, yuye, DU20 30 min, 1 time per day; 10 times a course of treatment	Clinical efficacy	Not reported

Zhao et al. (2018) [20]	80 EG: 40 CG: 40	EG : Male: 23 female: 17 Age: 61 ± 9 CG : Male: 25 female: 15 Age: 63 ± 8	The 4th national symposium on cerebrovascular diseases of the Chinese medical association Frenchay dysarthria rating scale	Acupoint: tongue acupoint: the root of the tongue, the middle of the tongue, the edge of the tongue, and the tip of the tongue, sublingual, jinjin, yuye; Body acupoint: GB20, REN23, GB20, ST40, SP6, KID3, yiming, gongxue, zhiqiang TA operation: prick on acupoint 30 min, 6 times per week; 2 weeks	Body acupoint: GB20, REN23, GB20, ST40, SP6, KID3, yiming, gongxue, zhiqiang 30 min, 6 d per week; 2 weeks	Clinical efficacy; Frenchay dysarthria rating scale	Not reported

Zhao et al. (2015) [21]	60 EG: 30 CG: 30	EG : Male: 26 female: 4 Age: 60.5 ± 9.8 CG : Male: 25 female: 5 Age: 61.3 ± 9.4	Scoring criteria of clinical neurological deficit in stroke patients Praat speech analysis software	Acupoint: tongue acupoint: Wai jinjin, yuye, lian quan, pang lianquan, juquan; Body acupoint: ST9, DU14, HT4, PC9, UB23, KID3, UB52 TA operation: oblique stab into the root of the tongue 2 times per day, 28 days	Body acupoint: PC6, DU26, HT5, GB20, GB12, SJ17, jinjin, yuye, posterior pharyngeal wall	NIHSS; clinical efficacy;	Not reported

*EG* experience group, *CG* control group, *LU7* Lieque*, KID6* Zhaohai*, HT5* Tongli*, PC6* Neiguan*, ST40* Fenglong*, SP6* Sanyinjiao*, LI4* Hegu*, LIV3* Taichong*, GB20* Fengchi*, REN23* Lianquan*, DU15* Yamen*, DU20* Baihui*. KID3* Taixi*, DU26* Shuigou*, SJ17* Yifen.

**Table 3:** Summary of acupoint location and operation methods.

Location of acupoints	Study	Acupoints
Lingual surface	Yu 2007	Xinxue, pixue
	Yuan 2020	Juhou
Sublingual	Chen 2021	Shengen
	Hao 2017	Jinjin, Yuye, beside REN23, above REN23
	He 2019	Shengen, zuoliang, zhimai
Both lingual surface and sublingual	Pan 2016	Juquan (Lingual surface); Haiquan (sublingual)
	Zhao 2015	Juquan (Lingual surface); wai jinjin, wai Yuye (sublingual)
	Zhao 2018	Lingual surface, tip, edge and root
Acupuncture operation	Study	
Oblique stabbing into the tongue root	Chen 2021, Hao2017, He2019, Pan 2016, Yuan2 020, Zhao 2015	
Twisting the needle body after stabbing	Chen 2021, Hao 2017, He 2019, Pan 2016, Yu 2007	
Acupoints pricking	Hao 2017, He 2019, Zhao 2018, Zhhao 2015	
Number of operating methods	Study	
One operation method	Yuan 2020, Zhao 2018, Yu 2007	
Two operation methods	Chen 2021, *Pan* 2016, Zhao 2015	
Three operation methods	Hao 2017, He 2019	

**Table 4:** Subgroup analysis results.

Subgroup	*N*	Sample size EG	CG	OR	95% CI	*P*	I^2^
Location of acupoints							
Lingual surface	3	97	90	3.17	(1.19, 8.45)	0.021	0%
Sublingual	3	94	94	3.74	(1.70, 8.22)	0.001	0%
Both Lingual surface and sublingual	3	95	95	3.82	(1.77, 8.24)	0.001	0%
Number of operating methods							
One operation method	4	137	130	2.97	(1.43, 6.15)	0.003	0%
Two operation methods	3	84	84	3.87	(1.67, 8.95)	0.002	0%
Three operation methods	2	65	65	4.71	(1.75, 12.66)	0.002	0%

## Data Availability

The research results will be published in public journals and the data can be shared by colleagues. No data were used to support this study.
